# Regulation of Interface Compatibility and Performance in Soft Magnetic Composites with Inorganic Insulation Layers by FePO_4_ Intermediate Transition Layer

**DOI:** 10.3390/molecules29225281

**Published:** 2024-11-08

**Authors:** Sanao Huang, Junjie Ma, Yang Liu, Hao He, Peisheng Lyu, Huaqin Huang, Bing Dai

**Affiliations:** 1 Anhui Key Laboratory of Low Carbon Metallurgy and Solid Waste Resource Utilization, Anhui University of Technology, Ma’anshan 243002, China; huangsanao@ahut.edu.cn (S.H.); ahutmajunjie@163.com (J.M.); ahutly1204@ahut.edu.cn (Y.L.); hehao@ahut.edu.cn (H.H.); lyu.peisheng@csu.edu.cn (P.L.); 2 School of Electrical and Information Engineering, Anhui University of Technology, Ma’anshan 243002, China; 3 School of Metallurgy and Environment, Central South University, Changsha 410083, China

**Keywords:** soft magnetic composites, phosphate, transition layer, comprehensive performance

## Abstract

In the fabrication of soft magnetic composites, the lattice mismatch between the inorganic insulation layer and the iron matrix often leads to the formation of cracks during the molding process, which significantly impairs the operational performance of the materials. Consequently, it is imperative to develop novel strategies for inorganic insulation coatings that offer high electrical resistivity and thermal stability and are less susceptible to cracking during formation. This paper presents a new structure for soft magnetic composites that incorporates FePO_4_ as an intermediate transition layer between the iron-based soft magnetic particles and the inorganic ceramic insulation layer. This configuration is designed to provide insulation coatings with superior voltage and thermal resistance, as well as high electrical resistivity. The research details the processes forming the FePO_4_ intermediate transition layer and the SiO_2_ insulation layer on the iron powder surface, along with their interaction mechanisms. An analysis comparing the scenarios with and without the FePO_4_ intermediate transition layer shows its beneficial impact on the magnetic properties and mechanical strength of the soft magnetic composites. Further investigations reveal that at a phosphoric acid concentration of 1 wt.%, the FePO_4_ layer significantly enhances the interface compatibility between the Fe powder matrix and the SiO_2_ insulation layer. Under these conditions, the Fe@ FePO_4_/SiO_2_ soft magnetic composites demonstrate outstanding overall performance: the saturation magnetization stands at 215.60 emu/g, effective permeability at 83.2, resistivity at 57.42 Ω·m, power loss at 375.0 kW/m^3^ under 30 mT/100 kHz, and radial compressive strength at 15.95 Kgf. These findings offer novel insights and practical approaches for advancing inorganic insulation coating strategies and provide vital scientific support for further enhancing the magnetic and mechanical properties of soft magnetic composites.

## 1. Introduction

Soft magnetic composites (SMCs) are extensively employed in various devices within power and electronics systems, favored for their high saturation magnetization, elevated resistivity, minimal eddy current losses, and outstanding thermal/magnetic isotropy in three dimensions [1,2,3]. The burgeoning fields of new energy vehicles and photovoltaic power generation have heightened demands for device miniaturization, high-frequency operation, and increased current capacity, thereby accelerating research into SMCs characterized by high permeability, low losses, stable performance at high frequencies, and superior DC bias attributes.

In high-frequency applications, core losses in SMCs predominantly stem from eddy current losses. These currents not only elevate component heating but also impair efficiency [4,5,6]. To mitigate core losses, particularly those from eddy currents, researchers have utilized insulation coatings on soft magnetic particles [7,8,9]. This method enhances the material’s overall resistivity and curtails eddy currents between particles. The insulation typically comprises a blend of organic and inorganic substances. Organic materials, like epoxy resins [10], acrylics [11], and polyurethane [12], offer excellent adhesion and flexibility but thermally decompose at temperatures nearing 300 °C, whereas SMCs necessitate annealing at 500–800 °C to alleviate internal stresses from high-pressure molding, thus reducing hysteresis losses [8,13,14]. The instability and heterogeneity of organic insulators at high temperatures restrict their use in high-temperature annealed SMCs, making inorganic insulators with superior thermal stability and high resistivity a focal point of research. Techniques such as simple ball milling to apply coatings of mica [15], kaolin [16], water glass [17], and various inorganic salts [18], and chemical methods like the sol–gel and wet chemical processes, can create uniform, adjustable-thickness ceramic insulation layers (e.g., SiO_2_, Al_2_O_3_, TiO_2_, Ce_2_O_3_, MnO) [19,20,21,22,23] and ferrimagnetic ferrites (e.g., NiZn, MnZn) [24,25] suitable for higher temperature treatments. Nonetheless, the substantial lattice mismatch between such inorganic coatings and the iron matrix can lead to cracking or detachment during subsequent high-pressure molding, significantly impacting the composites’ operational performance. In previous research, we explored the impacts of SiO_2_ insulation layer thickness on the microstructure and magnetic properties of FeSi powder and SMCs, discovering that thick SiO_2_ layers could induce interface stress, while excessively thin layers failed to meet required resistivity and thermal resistance standards [26,27]. Thus, advancing new inorganic insulation strategies that are highly resistive, thermally stable, and resistant to molding-induced cracking continues to be a pivotal direction for the development of SMCs.

Phosphates, recognized for their high electrical resistivity and stability, are frequently employed as coatings on iron-based materials through phosphate passivation, serving as insulation layers in SMCs. Numerous studies have shown that precisely controlling the thickness and structure of these phosphate coatings can substantially enhance the composites’ overall performance [28,29]. Our initial research indicates that the insulation layer formed by these salts not only secures mechanical anchoring with the iron substrate but also forms chemical bonds, ensuring superior adhesive strength. Moreover, the thermal expansion coefficients of the insulation layer and the iron substrate are comparable, reducing internal stress and interface delamination due to mismatch during thermal processing [28]. This compatibility helps maintain stable adhesion under cyclic temperature variations, significantly reducing the risk of cracks and delamination. Consequently, phosphates are poised to mitigate the issues of lattice mismatch and weak bonding between iron-based soft magnetic particles and inorganic ceramic insulation layers.

This study introduces a novel structure for SMCs, incorporating FePO_4_ as an inter-mediate transition layer between the iron-based magnetic particles and ceramic insulation layers, designed to provide a robust insulation strategy capable of withstanding high voltages, temperatures, and resistivity. Employing surface phosphatization and fluidized gas phase in situ deposition, Fe@ FePO_4_/SiO_2_ composite magnetic powders with an intermediate transition layer of FePO_4_ and an inorganic insulation layer of SiO_2_ were prepared. These powders were then pressed into iron-based SMCs. Detailed analyses of the composites’ microstructure and phase constitution provided insights into the formation processes of the FePO_4_ and SiO_2_ layers on the iron powder surface. Comparing these composites with others featuring only a single SiO_2_ insulation layer highlighted the crucial role of the FePO_4_ transition layer in enhancing magnetic performance and mechanical properties. These findings not only advance inorganic insulation coating techniques but also offer invaluable scientific insights into optimizing the magnetic performance of SMCs.

## 2. Results and Discussion

### 2.1. Formation Process of FePO_4_ Intermediate Transition Layer and SiO_2_ Insulation Layer on Surface of Fe Powder

**Figure 1:**
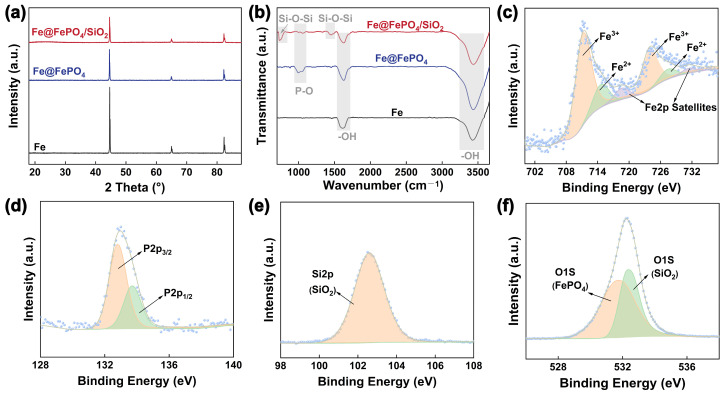
The detection results of raw Fe powder, Fe@FePO_4_ composite powder (with a H_3_PO_4_ concentration of 1.00 wt.%), and Fe@FePO_4_/SiO_2_ composite powder: (**a**) the XRD spectra; (**b**) the FTIR spectra. The XPS spectra of Fe@FePO_4_/SiO_2_ composite powder: (**c**) the Fe2p; (**d**) the P2p; (**e**) the Si2p; (**f**) the O1s.

To deeply explore the formation process and mechanism of the FePO_4_ intermediate transition layer and the SiO_2_ insulation layer on the surface of Fe powder, this study comprehensively employed XRD, FTIR, and XPS to characterize the raw Fe powder, Fe@ FePO_4_ composite powder (with a H_3_PO_4_ concentration of 1.00 wt.%), and Fe@ FePO_4_/SiO_2_ composite powder. Initially, XRD results (refer to Figure 1a) showed that the raw Fe powder exhibited three prominent diffraction peaks at 2θ angles of 44.7°, 65.0°, and 82.3°, corresponding to the (110), (200), and (211) planes of the body-centered cubic α-Fe phase, aligning with data from the International Crystal Structure Database (ICSD card no. 03-065-4899), confirming the high purity and crystalline integrity of the Fe powder. In the XRD spectra of the Fe@ FePO_4_ and Fe@ FePO_4_/SiO_2_ composite powders, besides the α-Fe phase peaks, no distinct peaks for FePO_4_ and SiO_2_ were detected, likely due to the formation of amorphous or nanocrystalline structures smaller than 5 nm during the phosphatization process [21]. Furthermore, the prior research indicated that the SiO_2_ layer formed via fluidized gas phase in situ deposition is amorphous [27]. The thin nature of the transition and insulation layers might also contribute to the non-detection by XRD.

The FTIR spectra further confirmed the presence of the FePO_4_ intermediate transition layer and SiO_2_ insulation layer (refer to Figure 1b). All samples displayed strong absorption peaks at 3447 cm^−1^ and 1635 cm^−1^ due to –OH vibrations from H_2_O molecules, attributed to the large specific surface area of the Fe powder facilitating slight oxidation to form Fe_2_O_3_ lattice vibrations. Compared to the raw Fe powder, the Fe@FePO_4_ composite powder showed distinct phosphate peaks at 1065 cm^−1^ [29]. The bands at 1065 cm^−1^ are attributed to the symmetric stretching vibrations of P–O bonds [30], respectively. In the Fe@FePO_4_/SiO_2_ composite powder, apart from the phosphate peaks, broad absorption peaks at 1049, 784, and 457 cm^−1^ were also noted, derived from the symmetric, asymmetric, and bending vibrations of Si–O–Si [19]. These findings not only corroborate the successful formation of the FePO_4_ layer through phosphatization but also confirm the subsequent coating of the SiO_2_ layer. Additionally, the observed weakening of Fe_2_O_3_ lattice vibrations on the powder surface post-passivation suggests an initial reaction of the H_3_PO_4_ with surface oxides before interacting with the underlying pure iron to form phosphate.

Finally, XPS spectroscopy has further analyzed the chemical structure and elemental states of the Fe@FePO_4_/SiO_2_ composite powder, as detailed in Figure 1c–f. Figure 1c reveals that the characteristic peaks at 710.3 eV and 712.8 eV correspond to the oxidation states of Fe^2+^ and Fe^3+^ [31], respectively, indicating the coexistence of these states within the composite powder. In Figure 1d, the P peaks due to spin–orbit splitting, with P2p_3/2_ and P2p_1/2_ peaks at 132.8 eV and 134.6 eV aligning with the phosphate structure [30]. The Si2p peak at 103.0 eV in Figure 1e is clearly associated with the SiO_2_ structure. The O1s spectrum in Figure 1f features two peaks at 531.8 eV and 532.3 eV, affirming the presence of PO_4_^3−^ and SiO_2_ [32]. These findings corroborate the FTIR spectral analysis presented in Figure 1b, verifying the successful formation of a FePO_4_ and SiO_2_ coating on the surface of water-atomized Fe powder through surface phosphatization and fluidized gas phase in situ deposition.

**Figure 2:**
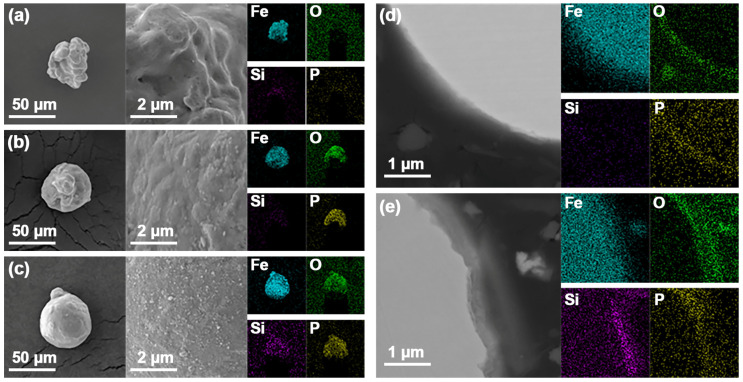
Surface morphology and elemental analysis: (**a**) raw Fe powder; (**b**) Fe@FePO_4_ composite powder (with H_3_PO_4_ concentration of 1.00 wt.%); (**c**) Fe@FePO_4_/SiO_2_ composite powder. Cross-sectional view and elemental analysis: (**d**) Fe@FePO_4_ composite powder (with 1.00 wt.% phosphoric acid); (**e**) Fe@FePO_4_/SiO_2_ composite powder.

Figure 2 illustrates the surface and cross-sectional morphologies and elemental distributions of the raw Fe powder, the Fe@FePO_4_ composite powder (with a H_3_PO_4_ concentration of 1.00 wt.%), and the Fe@FePO_4_/SiO_2_ composite powder. Figure 2a reveals that the raw Fe powder exhibits an irregular shape due to the high-pressure water jet impacting the liquid iron, causing the iron droplets to fragment irregularly; however, the powder surface remains relatively clean and smooth. Energy spectrum analysis shows that, apart from the uniformly distributed Fe element, a trace amount of O element also exists on the powder surface. Compared to the raw Fe powder, the Fe@FePO_4_ composite powder (shown in Figure 2b) features a more stable particle size and morphology, although the surface is rougher with a clearly visible coating layer. This analysis demonstrates a uniform distribution of Fe, P, and O elements, indicating robust integrity and adhesion of the FePO_4_ coating. Further treatment involving the Fe@FePO_4_/SiO_2_ composite powder (depicted in Figure 2c) results in the detection of Si along with Fe, P, and O, leading to a significantly thicker coating and the emergence of nano-scale clusters. Figure 2d,e depict the cross-sectional morphology and elemental distribution of the Fe@FePO_4_ and Fe@FePO_4_/SiO_2_ powders, respectively. The Fe@FePO_4_ sample distinctly shows a tri-layered structure comprising a white inner layer, a light gray intermediate layer, and a black outer layer. EDS mapping confirms that the inner layer primarily comprises Fe, the intermediate layer is enriched with Fe, P, and O, and the outer layer predominantly contains P and O. The Fe@FePO_4_/SiO_2_ sample introduces an additional dark gray layer between the intermediate and outer layers, with a significant enrichment of Si and O elements.

Based on the preliminary studies [33] and experimental observations of powder samples at various processing stages, supported by results from XRD, XPS, FITR, and SEM, we propose a hypothesis regarding the formation processes and bonding mechanisms of a FePO_4_ intermediate transition layer and a SiO_2_ insulation layer on the surfaces of Fe powders.

The first stage is as follows: H_3_PO_4_ engages in a chemical reaction with the iron oxide film on the Fe powder surface, followed by further reactions with the underlying pure Fe (outlined in Reactions (1) to (3)). This process facilitates the formation of robust chemical bonds of FePO_4_ molecules on the F powder surface, created through the interactions among Fe, O, and P elements, resulting in the synthesis of Fe@FePO_4_ composite powder. The reactions proceed as follows:

(1) $${Fe}^{2+}+{HPO}_{4}^{2-}\to {FeHPO}_{4}$$

(2) $${3Fe}^{2+}+{2PO}_{4}^{3-}\to {Fe}_{3}{\left({PO}_{4}\right)}_{2}$$

(3) $${4Fe}^{2+}+O_{2}+{4PO}_{4}^{3-}+{4H}^{+}\to {4FePO}_{4}+{2H}_{2}O$$

The second stage is as follows: TEOS serves as the precursor for the SiO_2_ layer. Before its introduction into the tube furnace by Ar gas, TEOS undergoes pre-decomposition in a liquid evaporator, producing Si(OH)(OC_2_H_5_)_3_ (Reactions (4) and (5)). This intermediate then participates in transesterification (Reaction (6)) and hydrolysis (Reaction (7)) with additional TEOS molecules, yielding O(Si(OC_2_H_5_)_3_)_2_.

(4) $$\mathrm{Si}{\left(OC_{2}H_{5}\right)}_{4}\to \mathrm{Si}\left(\mathrm{OH}\right){\left(OC_{2}H_{5}\right)}_{3}+C_{2}H_{4}$$

(5) $$\mathrm{Si}{\left(OC_{2}H_{5}\right)}_{4}+H_{2}O\to \mathrm{Si}\left(\mathrm{OH}\right){\left(OC_{2}H_{5}\right)}_{3}+C_{2}H_{5}\mathrm{OH}$$

(6) $$\mathrm{Si}\left(\mathrm{OH}\right){\left(OC_{2}H_{5}\right)}_{3}+\mathrm{Si}{\left(OC_{2}H_{5}\right)}_{4}\to C_{2}H_{5}\mathrm{OH}+O{\left(\mathrm{Si}{\left(OC_{2}H_{5}\right)}_{3}\right)}_{2}$$

(7) $$\mathrm{Si}\left(\mathrm{OH}\right){\left(OC_{2}H_{5}\right)}_{3}\to H_{2}O+O{\left(\mathrm{Si}{\left(OC_{2}H_{5}\right)}_{3}\right)}_{2}$$

The third stage is as follows: in the fluidized gas phase deposition system, Si(OH)(OC_2_H_5_)_3_ and fresh TEOS molecules are adsorbed onto the Fe@FePO_4_ composite powder surface and undergo thermal decomposition. The FePO_4_ layer provides both chemical functional groups and surface roughness, enhancing the adsorption and subsequent reactions of SiO_2_ precursor molecules. Moreover, the excellent interfacial compatibility between the amorphous FePO_4_ and SiO_2_ layers facilitates their integration. The series of reactions, including the thermal decomposition of ethoxy groups, transesterification between ethoxy and hydroxy groups, and dehydration condensation among hydroxy groups, continues until the ethoxy is fully converted into O-Si-O bonds, culminating in the formation of an amorphous SiO_2_ network (Reaction (8)).

(8) $$\mathrm{Si}{\left(OC_{2}H_{5}\right)}_{4}+\mathrm{Si}{\left(O-\mathrm{Si}-O\right)}_{3}\left(\mathrm{OH}\right)\to \mathrm{Si}O_{2}\left(D\right)+\mathrm{Si}(O-\mathrm{Si}-O){\left(OC_{2}H_{5}\right)}_{3}+C_{2}H_{5}\mathrm{OH}$$

Through a thorough analysis of these experimental observations, a deeper and more comprehensive understanding of the formation mechanisms and bonding characteristics of the FePO_4_ intermediate transition layer and SiO_2_ insulation layer on water-atomized iron powder surfaces is gained.

### 2.2. Impact of FePO_4_ Intermediate Transition Layer on Properties of Soft Magnetic Composites

**Figure 3:**
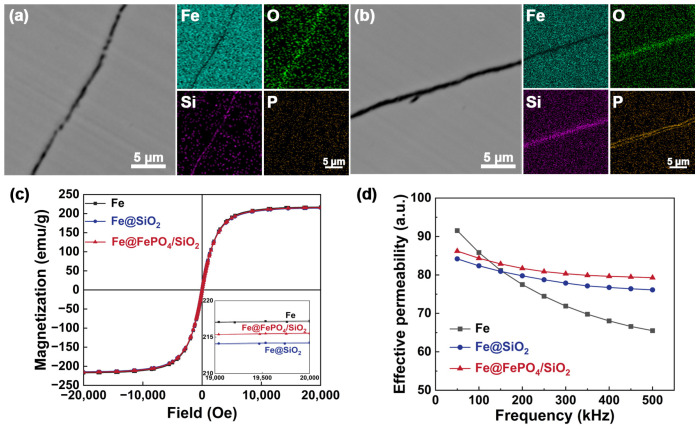
Cross-sectional view and elemental analysis: (**a**) Fe@SiO_2_ soft magnetic composites; (**b**) Fe@FePO_4_/SiO_2_ soft magnetic composites. Magnetic properties of Fe compacts, Fe@SiO_2_ soft magnetic composites, and Fe@FePO_4_/SiO_2_ soft magnetic composites: (**c**) hysteresis loops as function of frequency; (**d**) effective permeability as function of frequency.

Figure 3a,b show the cross-sectional views of the Fe@FePO_4_/SiO_2_ and Fe@SiO_2_ SMCs, respectively. SEM images reveal that the Fe@ FePO_4_/SiO_2_ SMCs maintain excellent interface compatibility between the FePO_4_ intermediate transition layer, the Fe powder, and the SiO_2_ insulation layer, characterized by continuity and an absence of visible gaps or fractures. Conversely, the Fe@SiO_2_ SMCs display a heterogeneous distribution of Fe powder relative to the SiO_2_ insulation layer, featuring noticeable cracks and voids. This disparity likely stems from significant differences in thermal expansion coefficients and elastic moduli, resulting in stress concentrations at the interface.

At 25 °C, hysteresis loops were recorded for the Fe compacts, Fe@SiO_2_ SMCs, and Fe@ FePO_4_/SiO_2_ SMCs to evaluate their magnetization characteristics. As depicted in Figure 3c, all samples showed hysteresis loops that typify soft magnetic qualities, such as ease of magnetization, high saturation magnetization, and low coactivity. Comparative studies of the saturation magnetization strengths within a magnetic field range of 19,000–20,000 Oe (refer to Figure 3c inset) indicate that saturation magnetization strength in SMCs correlates directly with the volumetric ratio of the internal ferromagnetic phase [34]. The integration of non-magnetic FePO_4_ and SiO_2_ layers increases the separation among internal magnetic particles, thus reducing the volume ratio of Fe powder and raising that of non-magnetic materials. Hence, the saturation magnetization strength of the Fe compacts (217.2 emu/g) surpasses that of the Fe@SiO_2_ SMCs (214.3 emu/g) and Fe@ FePO_4_/SiO_2_ SMCs (215.6 emu/g). In fact, although Fe@FePO_4_/SiO_2_ SMCs have an additional non-magnetic interlayer compared to Fe@SiO_2_ SMCs, the existence of the intermediate layer enhances the interface bonding between Fe powder and SiO_2_, improves structural integrity, and reduces local magnetic domain distortion caused by stress concentration. Therefore, the porosity is reduced and the number of magnetic domains per unit volume is increased. Consequently, the M_s_ of Fe@FePO_4_/SiO_2_ SMCs is improved.

Figure 3d illustrates how the effective permeability of various materials varies with frequency. Between 1 and 100 kHz, Fe compacts exhibit a significantly higher effective permeability (91.5) compared to Fe@SiO_2_ SMCs (84.2) and Fe@FePO_4_/SiO_2_ SMCs (86.1). The difference primarily arises from the introduction of non-magnetic FePO_4_ as an intermediate transition layer and SiO_2_ as an insulation layer. These layers create physical barriers between magnetic particles, diminishing their interactions, altering magnetic domain distribution and structure, and impeding the smooth movement of domain walls [35,36]. As testing frequencies increase, all samples demonstrate a declining trend in effective permeability. Notably, the permeability of the Fe compacts starts to decrease above 150 kHz, whereas for Fe@SiO_2_ SMCs and Fe@FePO_4_/SiO_2_ SMCs, significant reductions occur beyond 350 kHz and 300 kHz, respectively. This behavior is attributed to the high-resistivity SiO_2_ insulation layer, which limits domain wall motion and magnetic coupling among adjacent particles, effectively curbing eddy and induced currents, thus reducing overall current dissipation. Moreover, interface compatibility among different materials within the SMCs significantly affects their performance. Introducing FePO_4_ as an intermediate layer strengthens the bond at the interfaces with Fe powder and SiO_2_, enhancing structural integrity and reducing local distortions in magnetic domains due to stress concentration [37,38]. This modification allows magnetic domains to respond more effectively to external magnetic fields, thereby enhancing effective permeability and improving frequency stability. Consequently, Fe@FePO_4_/SiO_2_ SMCs show superior stability and adaptability across an extensive frequency range.

**Figure 4:**
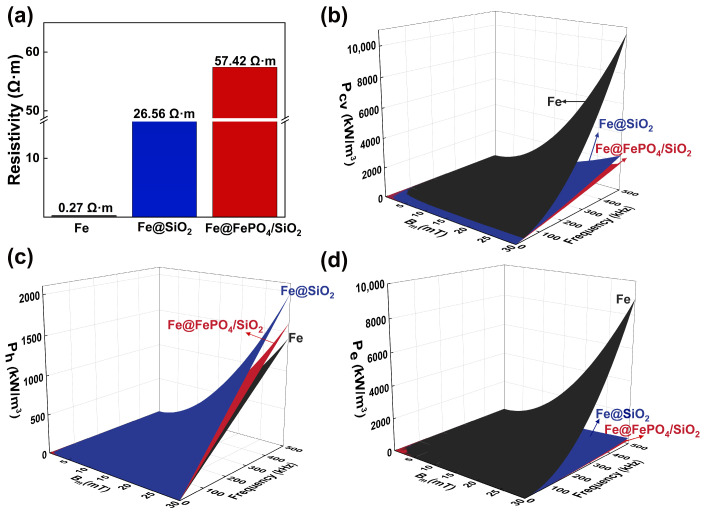
Magnetic property for Fe compacts, Fe@SiO_2_ soft magnetic composites, and Fe@FePO_4_/SiO_2_ soft magnetic composites: (**a**) resistivity; (**b**) total core loss; (**c**) hysteresis loss; (**d**) eddy current loss.

Resistivity is a crucial parameter for evaluating the electrical insulation characteristics of SMCs, significantly influencing the material’s energy losses. In this study, a high dielectric constant SiO_2_ insulation layer (with a resistivity range of 10^12^ to 10^15^ Ω·m) was introduced into SMCs [39,40]. This addition not only enlarges the gaps between the Fe powder matrices but also disrupts the continuous, dense, and conductive network formed by the magnetic powders, thus posing significant barriers to current flow. Additionally, the SiO_2_ insulation layer on the surface of the Fe powder matrix induces interlayer and vibrational scattering, further impeding current propagation. As depicted in Figure 4a, the resistivity of Fe@FePO_4_/SiO_2_ SMCs reaches 57.42 Ω·m, in stark contrast to the 26.56 Ω·m of Fe@SiO_2_ SMCs. When compared with the resistivity of Fe compacts (0.27 Ω·m), these materials exhibit resistivity increases in two orders of magnitude, respectively. The introduction of a FePO_4_ intermediate transition layer between the Fe powder matrix and the SiO_2_ insulation layer markedly enhances the resistivity. This effect arises partly from the inherent high resistivity of the FePO_4_ layer (10^5^ to 10^8^ Ω·m) [40], which substantially elevates the overall resistivity of the material. Moreover, this layer improves the interface matching between the Fe powder and the SiO_2_ insulation layer, thereby enhancing interface uniformity and integrity and minimizing the formation of conductive pathways.

Figure 4b illustrates the total core loss (*P_cv_*) in Fe compacts, Fe@SiO_2_ SMCs, and Fe@FePO_4_/SiO_2_ SMCs varies with test frequency and magnetic flux density. With increases in both frequency and magnetic flux density, the rate of magnetic domain rearrangement must accelerate, resulting in greater energy dissipation, more frequent domain walls movement, and enhanced damping effects [41]. Consequently, the *P_cv_* for both the Fe compacts and Fe-based SMCs rises with frequency. Specifically, under testing conditions of 30 mT and 100 kHz, the Fe@FePO_4_/SiO_2_ SMCs demonstrate the lowest total loss at 375.0 kW/m^3^, which is 43.3% lower than the Fe compacts at 662.5 kW/m^3^ and 13.3% less than the Fe@SiO_2_ composites at 432.6 kW/m^3^.

The components of total loss (*P_cv_*) in SMCs—hysteresis (*P_h_*), eddy current (*P_e_*), and residual losses (*P_r_*)—are delineated in Equation (9). *P_h_*, the energy expended during magnetization and demagnetization in alternating magnetic fields, is proportional to frequency and exponentially dependent on the maximum magnetic induction (*B_m_*), as per Equation (10). *P_e_*, resulting from internally induced eddy currents that produce heat and energy loss, scales with the square of the frequency (*f*) and exponentially with the *B_m_*, according to Equation (11). *P_r_*, attributed to internal stresses, grain boundaries, and impurities causing uneven magnetization, are significant only under extremely low or high frequencies and are generally negligible in medium to high-frequency applications in soft magnetic materials [42,43,44,45,46].

(9) $$P_{cv}=P_{h}+P_{e}+P_{r}$$

(10) $$P_{h}=C_{h}B_{m}^{\alpha }f$$

(11) $$P_{e}=\frac{C_{e}B_{m}^{x}f^{2}}{\rho_{s}}$$

Equations (10) and (11) denote that *C_h_* and *C_e_* are the coefficient for *P_h_* and *P_e_*, *ρ_s_* represents the resistivity of the SMCs, and the fitting coefficient α, typically between 1.6 and 2.2 for the softest magnetic materials. Utilizing this theoretical framework, a nonlinear regression analysis of *P_h_* and *P_e_* at various frequencies was performed, with findings presented in Figure 4c,d.

As depicted in Figure 4c, the P_h_ observed in Fe compacts is markedly lower than in Fe@SiO_2_ SMCs and Fe@FePO_4_/SiO_2_ SMCs. This difference stems from the inclusion of non-magnetic FePO_4_ intermediate transition layers and SiO_2_ insulation layers, which cause an uneven magnetic field distribution and extend the magnetic path length, enhancing energy dissipation when the field transitions between magnetic and non-magnetic phases. Moreover, the SiO_2_ insulation layer in Fe@SiO_2_ SMCs is thicker than the total thickness of the FePO_4_ intermediate transition layer and SiO_2_ insulation layer in the Fe@FePO_4_/SiO_2_ SMCs. This discrepancy reduces the volume of ferromagnetic material and increases the irregularity in the rotation of magnetic moments, thereby augmenting energy loss during magnetic domain wall motion. Consequently, Fe@SiO_2_ SMCs exhibit higher hysteresis losses compared to Fe@FePO_4_/SiO_2_ SMCs. As shown in Figure 4d, the eddy current losses in Fe@FePO_4_/SiO_2_ SMCs are slightly lower than those in Fe@SiO_2_ SMCs, both significantly below those of Fe compacts. The intermediate transition and insulation layers act as barriers to electrical current formation, akin to adding resistors between magnetic particles, which complicates current flow and reduces both the magnitude and loss from eddy currents. In conclusion, incorporating FePO_4_ as an intermediate transition layer enhances the overall magnetic properties of Fe@SiO_2_ SMCs, offering broader frequency applicability, increased resistivity, and reduced energy consumption, thereby suggesting promising advancements in the field of SMCs.

**Figure 5:**
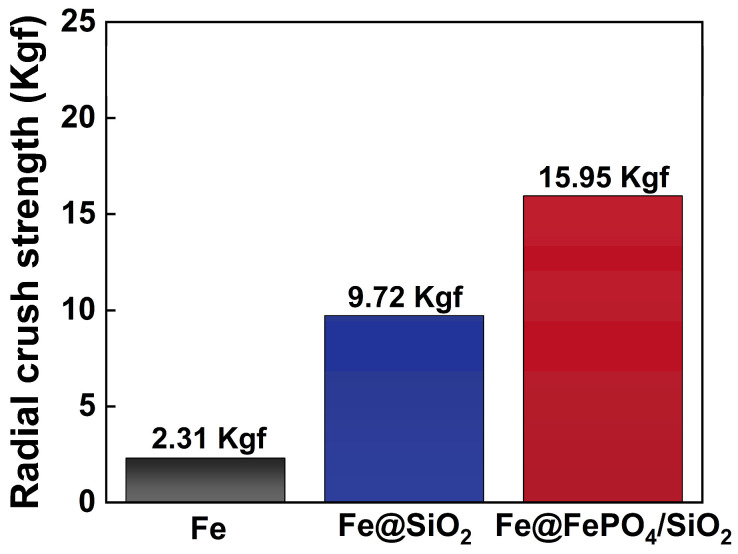
Radial compressive strengths of Fe compacts, Fe@SiO_2_ soft magnetic composites, and Fe@FePO_4_/SiO_2_ soft magnetic composites.

This study conducted radial crush tests on Fe compacts, Fe@SiO_2_ SMCs, and Fe@FePO_4_/SiO_2_ SMCs to assess and compare their radial compressive strengths. As depicted in Figure 5, annular samples were subjected to radial pressure to measure their strength. The Fe compacts demonstrated a radial compressive strength of 2.31 Kgf. Coating the Fe powder with SiO_2_, forming Fe@SiO_2_ SMCs, significantly boosted the strength to 9.72 Kgf, a notable enhancement primarily due to TEOS role during thermal decomposition. In this process, dehydration and condensation reactions between –OH groups formed a mesh-like SiO_2_ structure, markedly strengthening the powder bonds. Additionally, introducing FePO_4_ as an intermediate transition layer to produce Fe@FePO_4_/SiO_2_ SMCs, with FePO_4_ enhancing both the bond to the iron powder base and the interface with the SiO_2_ insulation layer, improves the material’s structural integrity and stability. The radial compressive strength of the Fe@FePO_4_/SiO_2_ SMCs was further elevated to 15.95 Kgf, highlighting the beneficial impact of these structural modifications on material performance.

### 2.3. Impact of H_3_PO_4_ Concentration on Structural and Magnetic Characteristics of SMCs

**Figure 6:**
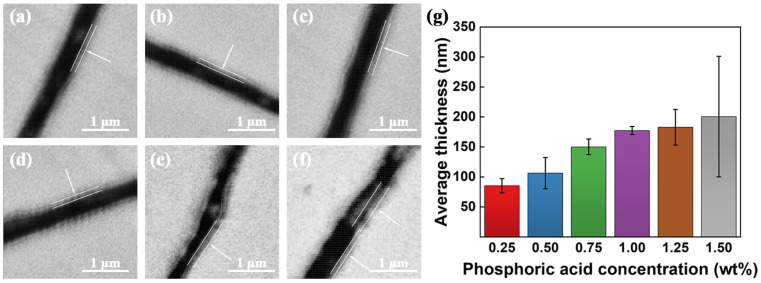
Electron backscatter diffraction images of Fe@FePO_4_/SiO_2_ soft magnetic composites at various phosphoric acid concentrations: (**a**) 0.25 wt.%, (**b**) 0.50 wt.%, (**c**) 0.75 wt.%, (**d**) 1.00 wt.%, (**e**) 1.25 wt.%, (**f**) 1.50 wt.%, (**g**) the trend in thickness of the FePO_4_ intermediate transition layer as function of phosphoric acid concentration.

This research delves into the influence of H_3_PO_4_ concentration on the microstructural characteristics of the FePO_4_ intermediate transition layer and the SiO_2_ insulation layer within Fe@FePO_4_/SiO_2_ SMCs. Electron backscatter diffraction imagery of these composites at varying concentrations of H_3_PO_4_ (refer to Figure 6) reveals a distinct stratification into a three-layer core–shell structure: the base matrix of Fe powder appears white, the FePO_4_ intermediate transition layer is light gray, and the SiO_2_ insulation layer is dark gray. Measurements and analysis of these layers indicate that at a H_3_PO_4_ concentration of 0.25 wt.% (Figure 6a), the average thicknesses of the FePO_4_ and SiO_2_ layers were approximately 85.3 nm ± 11.8 nm and 214.3 nm ± 20.1 nm, respectively. With increases in H_3_PO_4_ concentration to 1.00 wt.% (Figure 6d), the FePO_4_ layer thickened significantly to 177.4 nm ± 6.8 nm, while the SiO_2_ layer maintained a consistent thickness of about 200 nm. At higher concentrations of 1.25 wt.% (Figure 6e) and 1.5 wt.% (Figure 6f), the FePO_4_ layer’s thickness escalated further to 182.9 nm ± 29.7 nm and 200.6 nm ± 100.5 nm. Conversely, the thickness of the SiO_2_ layer diminished and the uniformity decreased, likely due to the irregular thickening of the FePO_4_ layer caused by high H_3_PO_4_ levels. This led to excessive crosslinking and hardening, reducing the material’s ductility [47], which in turn, prompted cracking within the SiO_2_ layer and interfered with its deposition and growth. Figure 6g illustrates the variations in thickness of the FePO_4_ layer as a function of H_3_PO_4_ concentration.

Table 1 displays the key performance parameters of Fe@FePO_4_/SiO_2_ SMCs prepared at varying H_3_PO_4_ concentrations, including saturation magnetic induction, effective permeability, resistivity, total core loss, and radial compressive strength. Notably, an increase in H_3_PO_4_ concentration from 0.25 wt.% to 1.25 wt.% correlates with a decrease in both saturation magnetic induction and effective permeability. This trend is primarily due to the thickening of the intermediate transition layer within FePO_4_, which extends the separation between the magnetic particles and diminishes their magnetic coupling—crucial for magnetic performance. The increase in layer thickness weakens the interparticle interactions, adversely affecting the saturation magnetic induction and effective permeability. Concurrently, the observed patterns of an initial increase followed by a decrease in resistivity and radial compressive strength, along with the initial improvement and subsequent deterioration in loss, are intimately tied to the microstructural features, thickness, and uniformity of the intermediate transition layer and the insulation layer. A uniformly thick and appropriate intermediate transition layer and insulation layer can effectively impede conductive pathways, enhancing the material’s resistivity and reducing eddy current loss, thereby benefiting its electromagnetic properties. However, excessive H_3_PO_4_ concentration leads to uneven layer thickness and the emergence of cracks within the intermediate transition layer, diminishing resistivity, compromising mechanical strength, and escalating losses, thus detrimentally affecting the overall material performance. When the concentration reaches 1.00 wt.%, Fe@FePO_4_/SiO_2_ SMCs exhibit optimal magnetic properties, achieving peak values in saturation magnetic induction and resistivity, with effective permeability and total loss also at their best. This underscores the importance of meticulously controlling H_3_PO_4_ concentration during material preparation, as mastering these microstructural parameters is vital for advancing the performance of SMCs in future applications and material innovations.

## 3. Materials and Methods

In this research, spherical Fe powder with over 99.5% purity, sourced from Anshan Metallurgical Powder Co., Ltd., (Anshan, China) served as the base magnetic particles. The chemical reagents included reagent-grade phosphoric acid (H_3_PO_4_) and acetone (CH_3_COCH_3_, 99.5% purity), both acquired from Shanghai Macklin Biochemical Technology Co., Ltd. (Shanghai, China); tetraethyl orthosilicate (TEOS, (C_2_H_5_O)_4_Si), provided by National Medicine Chemical Reagents Co., Ltd. (Shanghai, China); and lithium stearate along with silicone resin (REN50, containing more than 50 wt% solid), produced by Guangdong Boxing New Material Technology Co., Ltd. (Guangzhou, China). Deionized water from the lab’s purification system was employed in the experiments.

**Figure 7:**
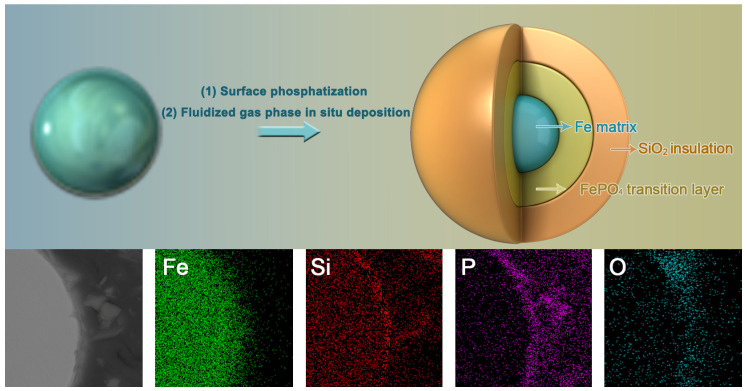
Preparation process and cross-sectional view of soft magnetic composite powder coated with FePO_4_ intermediate transition layer and SiO_2_ insulation layer.

The fabrication of Fe@FePO_4_/SiO_2_ SMCs incorporating a phosphate transition layer proceeded through three principal steps, as shown in Figure 7 and as follows:

(I) Surface phosphatization of Fe powder: A solution of H_3_PO_4_ mixed with 5 mL of acetone was stirred for 1 min, and 20 g of Fe powder was added. After 30 min of mechanical stirring, the mixture was washed thrice with anhydrous ethanol and dried at 80 °C for 2 h in a vacuum oven, yielding phosphatized Fe powder. Various ratios of H_3_PO_4_ to Fe powder (0.25, 0.50, 0.75, 1.00, 1.25, 1.5 wt.%) were explored to study the effects on the phosphatization process and the resultant phosphate transition layer.

(II) Fluidized gas phase in situ deposition of the SiO_2_ insulation layer: We placed 15 g of the phosphatized Fe powder on a stainless steel mesh (30 mm diameter, 5 μm pore size) within our custom-designed fluidized gas phase in situ deposition apparatus. High-purity argon gas was fed at 200 mL/min to maintain the powder in a fluidized state. At 650 °C, vaporized TEOS (evaporated at 150 °C) was introduced at 3 mL/min. After 1 h of deposition under standard atmospheric conditions, Fe@FePO_4_/SiO_2_ composite powder was obtained.

(III) Cold pressing to form SMCs: A mixture of Fe@FePO_4_/SiO_2_ composite powder, 0.5wt% lithium stearate, and 1wt% silicone resin was compressed under 1200 MPa into ring-shaped samples (12.6 mm outer diameter, 7.60 mm inner diameter, 4.81 mm thickness). The samples were then annealed at 500 °C in an argon atmosphere for one hour to alleviate internal stresses.

Moreover, a comparative experiment using Fe@SiO_2_ composite powder, prepared with the same coating technique, was conducted to assess the impact of the FePO_4_ intermediate transition layer on the properties of SMCs.

The phase composition of the powder samples was characterized using X-ray diffraction (XRD, Panacea Empyrean Sharp Shadows, The Netherlands) with a 2θ scan range of 20–90°, scan speed of 2° per minute, and a step size of 0.01 Å using Cu Kα radiation. Fourier transform infrared spectroscopy (FTIR, Thermo Nicolet IS, Thermo Fisher, Waltham, MA, USA) was utilized to examine the insulation layer’s chemical structure within the powder samples. Using X-ray photoelectron spectroscopy (XPS, Thermo Scientific K-Alpha, Waltham, MA, USA), with an Al Kα radiation source spanning a binding energy range of 0–1300 eV, the compositions and chemical states of the original Fe powder, Fe@FePO_4_ composite powder, and Fe@FePO_4_/SiO_2_ composite powder were determined. Surface/section morphology and elemental distribution of the powder and subsequent soft magnetic composites were analyzed using a field emission scanning electron microscope (FESEM, BD-S5500, Hitachi, Tokyo, Japan) equipped with an energy dispersive spectrometer (EDS).

Vibrating sample magnetometry (SQUID-VSM, LakeShore7404, Westerville, OH, USA) was performed to measure the hysteresis loops of Fe compacts, Fe@SiO_2_ SMCs, and Fe@FePO_4_/SiO_2_ SMCs at 25 °C in a field range of −20,000 to 20,000 Oe with a step size of 50 Oe. The samples were sized at 3 mm × 3 mm × 1 mm for these measurements, with saturation magnetization expressed in emu/g. The bulk resistivity was assessed using a four-probe resistivity analyzer (ST2722-SZ, Suzhou Crystal Electronics Co., Ltd., Suzhou, China) under a 1 MPa pressure, achieving an accuracy of 0.01 Ω·cm. An LCR meter (HIOKI IM3570A988-06, Hioki, Japan) was used to measure inductance at frequencies from 10 kHz to 10 MHz with a test voltage of 1 V and total coil turns of 40, calculating the effective permeability. The mid-to-high frequency losses of all SMCs samples were quantified using a B-H analyzer (SY-8258, Iwatsu, Tokyo, Japan).

## 4. Conclusions

In this research, the Fe@FePO_4_/SiO_2_ soft magnetic composite powders, featuring a FePO_4_ intermediate transition layer and a SiO_2_ insulation layer, were successfully synthesized through phosphatization and fluidized gas phase in situ deposition. These powders were then pressed into high-performance iron-based soft magnetic composites. The SiO_2_ insulation layer exhibits excellent thermal resistance and forms a three-dimensional disordered network, ensuring effective insulation. The FePO_4_ intermediate transition layer, facilitating chemical interactions among iron, oxygen, and phosphorus, creates stable bonds that enhance surface roughness and promote adsorption and reaction of the SiO_2_ precursor molecules, improving the interface compatibility with the Fe powder and bolstering structural integrity and compressive strength. The phosphoric acid concentration critically affects the thickness and microstructure of these layers, thus tuning the composites’ magnetic properties, resistivity, and losses. At 1.00 wt.% phosphoric acid, the Fe@FePO_4_/SiO_2_ soft magnetic composite achieves optimal magnetic performance with a saturation magnetization of 215.60 emu/g, an effective permeability of 83.2, a resistivity of 57.42 Ω·m, losses of 375.0 kW/m^3^ at 30 mT and 100 kHz, and a high compressive strength of 15.95 Kgf. These results underscore the significant potential of iron phosphate as an intermediate layer, enhancing the magnetic characteristics of iron-based composites. Future studies will investigate the extension of this approach to other soft magnetic materials like FeSi and FeNi.

## Figures and Tables

**Table 1:** Key performance parameters of Fe@FePO_4_/SiO_2_ soft magnetic composites at varying phosphoric acid concentrations.

H_3_PO_4_ Concentration	Radial Compressive Strength (Kgf)	M_s_ (emu/g)	μ_e_ (300 kHz)	*ρ* (Ω·m)	*P_cv_* (kW/m^3^)		
					10 mT, 100 kHz	10 mT, 300 kHz	10 mT, 500 kHz
0.25 wt%	10.13	216.13	79.1	41.35	65.73	214.09	462.93
0.50 wt%	12.22	215.96	80.8	47.13	459.04	183.95	313.56
0.75 wt%	13.87	215.77	81.4	51.68	51.41	161.92	285.24
1.00 wt%	15.95	215.60	83.2	57.42	43.69	138.64	243.08
1.25 wt%	13.57	210.69	78.3	50.86	47.80	159.41	279.36
1.50 wt%	11.72	209.18	71.6	42.98	51.33	173.29	345.17

## Data Availability

The raw data files can be downloaded and viewed at the following figshare https://figshare.com/articles/dataset/original_data/27233604?file=49822038 (accessed on 7 November 2024).
